# Supplementary material for: Extreme Food Insecurity and Malnutrition in Haiti: Findings from a Population-Based Cohort in Port-au-Prince, Haiti
Source: Nutrients. 2022 Nov 17;14(22):4854. doi: 10.3390/nu14224854 (PMC9695391; doi:10.3390/nu14224854)
Supplement: Supplementary file 1 [file nutrients-14-04854-s001.zip › nutrients-1964708-supplementary.pdf]

Supplementary Materials: The following supporting information can be downloaded at: https://www.mdpi.com/article/10.3390/nu14224854/s1, Figure S1: Flow Diagram; Figure S2: Comparison of USDA Household FIS categories and Modified Household FIS Categories, Figure S3: Proposed causal relationship between FIS and malnutrition.

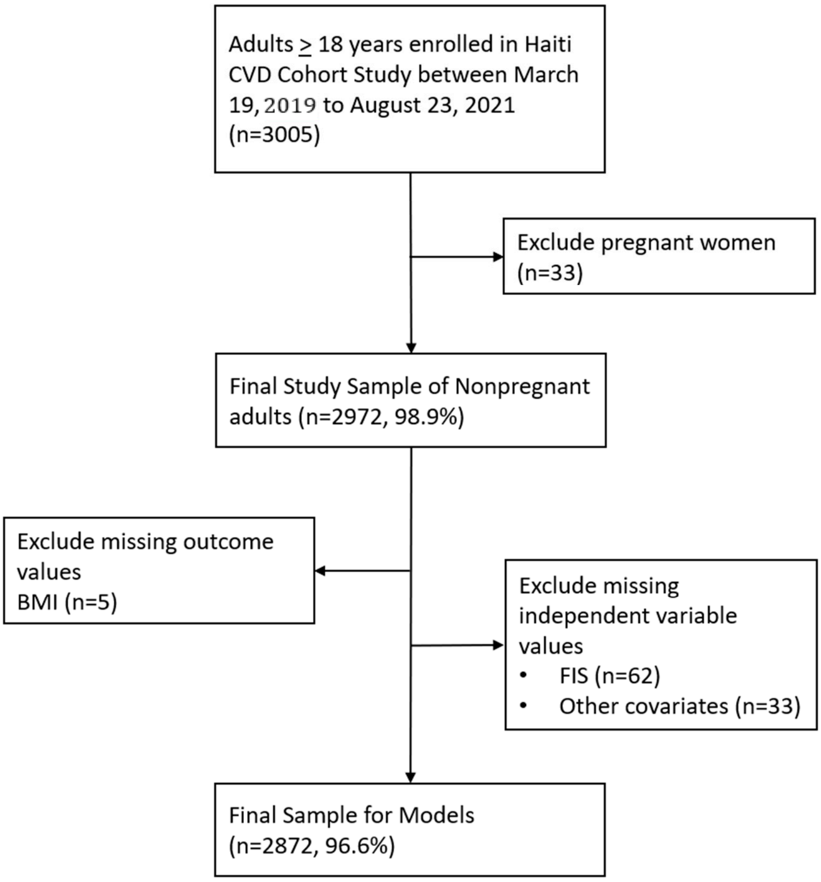

Figure S1. Flow diagram 2019

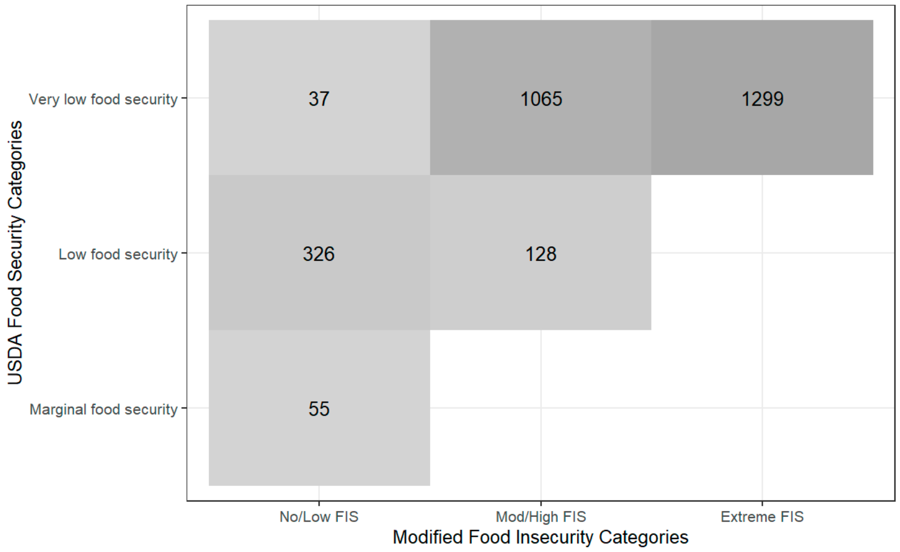

Figure S2. Comparison of USDA Household FIS categories[a] and Modified Household FIS categories[b]. FIS=food insecurity. [a] FIS categories base on USDA Household Food Security scale. Marginal food security (0-1), low food security (2-4) and very low food security (5-6). [b] FIS categories based on the modified version of the Six-Item Short Form of the Household Food Security Scale. No/low FIS (0-2), moderate/high FIS (3-4), and extreme FIS (5-6).

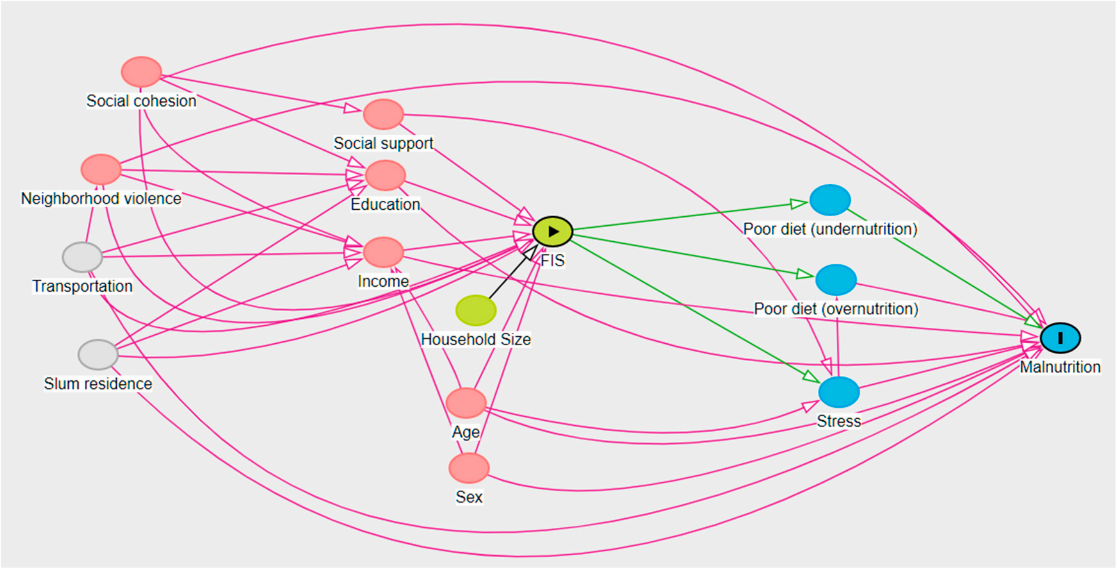

Figure S3. Proposed causal relationship between food insecurity (FIS) and malnutrition. Figure created using daggity.net.
